# Surgical myectomy: Rationale and personalized technique

**DOI:** 10.21542/gcsp.2018.35

**Published:** 2018-08-12

**Authors:** Hamood N. Al Kindi, Magdi H. Yacoub

**Affiliations:** 1 Aswan Heart center, Aswan Governate, Egypt; 2 Department of Cardiothoracic Surgery, Sultan Qaboos University Hospital, Muscat, Sultanate of Oman; 3 Department of Cardiac Surgery, Royal Brompton and Harefield NHS Trust, London, UK

## Abstract

Septal myectomy is currently the gold standard treatment for symptomatic patients with hypertrophic cardiomyopathy. The procedure needs to be tailored and performed in a personalized fashion, taking into consideration the anatomical spectrum of this disease. The procedure needs to address the various components that contribute to the clinical and pathological picture of this disease including, the fibrous trigones, accessory tissues, papillary muscles, mitral valve and myocardial bridges. The operation can be performed with very low mortality and morbidity in high-volume experienced centers with predictable excellent short and long-term outcomes. There is a need for broadening the experience of this procedure to the rest of the world and for future development of new enhanced precision imaging and surgical tools.

## Introduction

Hypertrophic cardiomyopathy (HCM) is the most common genetic cardiac disorder with a prevalence of 1:500 in the adult general population. LVOT obstruction is a common feature of the disease and plays an important role in the clinical outcome, both in terms of symptoms and longevity^1,2^. Septal myectomy remains the gold standard therapy for HCM patients who are symptomatic with high LVOT gradients^3^. The results of this procedure have vastly improved over time and it is now performed with low mortality and morbidity in experienced surigical centers. The results of this operation depend on thorough understanding of the sophisticated function of the LVOT and its derangements in HCM. We here describe the functional and surgical pathology of the left ventricular outflow tract (LVOT), followed by the key elements of the restorative tailored operation.

**Figure 1.**
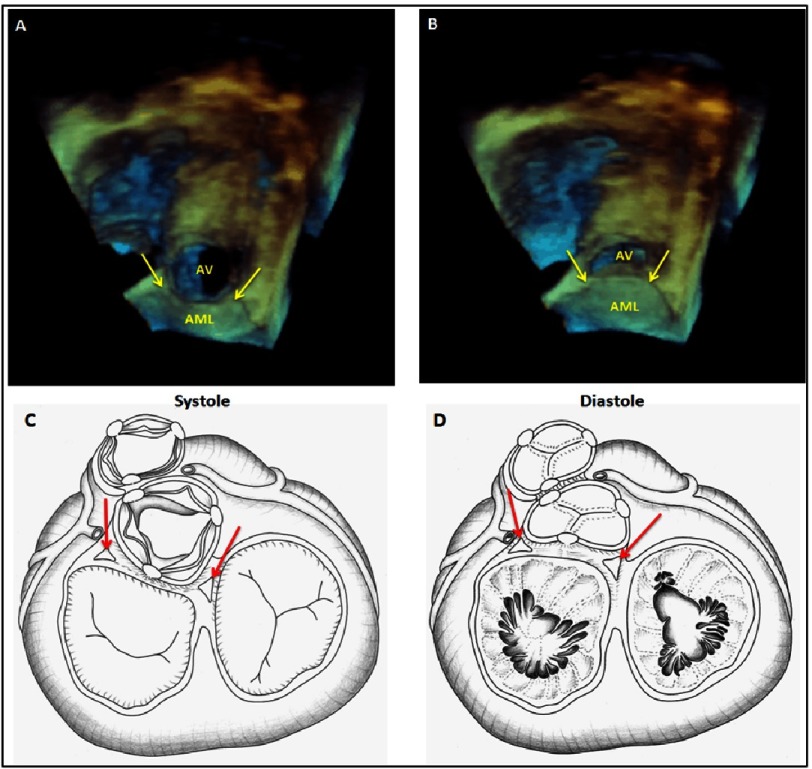
Both the LVOT and the mitral valve annulus share a common opening with optimized flow via dynamism of the aorto-mitral curtain both in systole (a,c) and diastole (b,d). LVOT, left ventricular outflow tract; AML, anterior mitral leaflet; AV, aortic valve^4^.

### The anatomy of the LVOT and its abnormalities in HCM

The aortic root and left ventricular outflow tract (LVOT ) occupy a central location in the heart and connect it to the rest of the arterial system^1,4,5^. The LVOT shares the same orifice with the mitral valve (Figure 1). The aortic and mitral orifices are connected by the sub-aortic curtain, which is a dynamic structure that moves during the cardiac cycle to allow the aortic orifice to enlarge during systole and the mitral valve during diastole. This requires a hinge mechanism and it is enabled by the two important components of the fibrous skeleton of the heart, termed the right and left fibrous trigones. The right fibrous trigone is located at the junction of the sub-aortic membrane to the membranous inter-ventricular septum and it is closely related to the septal leaflet of the tricuspid valve and the atrioventricular bundle of His. The left fibrous trigone is located at the attachment of the sub-aortic curtain to the muscular inter-ventricular septum at the postero-lateral angle of the LVOT, and the antero-lateral commissure of the mitral valve.

Abnormalities of the left fibrous trigone are more common in HCM patients than those of the right. The fusion of the left fibrous trigone prevents the subaortic curtain from moving backwards during systole and contribute significantly to systolic anterior motion (SAM) of the mitral valve and LVOT obstruction.

### The mitral valve and subvalvular apparatus in HCM

**Figure 2.**
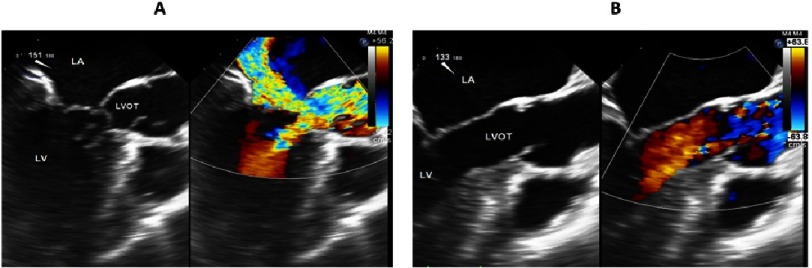
Pre- and post-myectomy TEE images. (A) Trans-esophageal echocardiography (mid-esophageal long axis view) showing the septal bulge of HOCM covered by a glistening endocardium denoting fibrosis, severe SAM with accessory chord attached to the interventricular septum. (B) Post-operative trans-esophageal echocardiography (mid-esophageal long axis view) of the same patient, showing laminar flow in the LVOT after the resection of the septal bulge and the accessory chord with the elimination of SAM. (TEE, transesophageal echocardiography; SAM, systolic anterior motion; LVOT, left ventricular outflow tract; LA, left atrium; LV, left ventricle)

**Figure 3.**
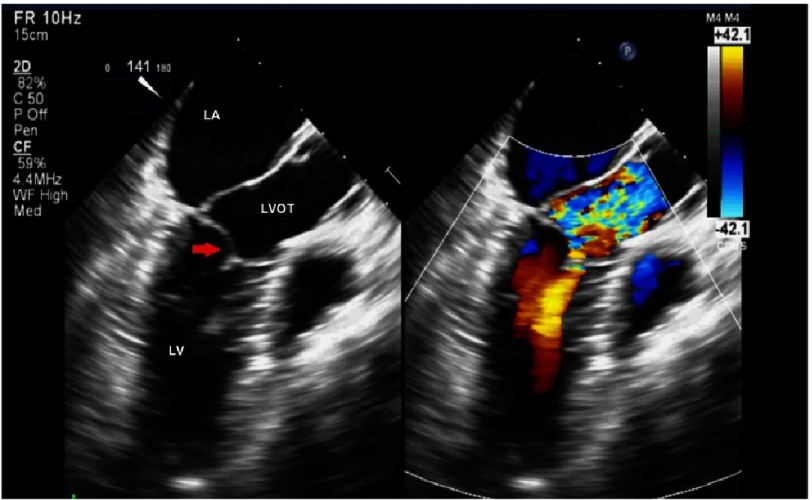
Systolic anterior motion of the posterior leaflet (PAM) in hypertrophic cardiomyopathy with flow acceleration in the LVOT. (Red arrow, posterior leaflet; LA, left atrium; LV, left ventricle; LVOT, left ventricular outflow tract)

The mitral valve contributes to subaortic obstruction in patients with HCM via systolic anterior motion. The structural abnormalities of the mitral valve in HCM are seen prior to the development of septal hypertrophy or flow acceleration in the LVOT. SAM is produced by the paradoxical movement of the anterior leaflet of the mitral valve, or occasionally by the posterior leaflet (PAM), toward the septum that produce pressure gradient across the LVOT and induce mitral valve regurgitation (Figures 2 and 3)^6,7^.

SAM was thought to be caused by Venturi effect that results in a suction effect of the mitral valve toward the ventricular septum. Another mechanism of SAM is due to elongated leaflets or displaced papillary muscles altering the coaptation point between the anterior and posterior leaflet of the mitral valve. This situation creates a residual portion of the anterior leaflet which will be pushed by the ejection of blood during systole. This explains why obstructive SAM can accompany modest septal hypertrophy when excessive or segmental leaflet elongation and papillary muscle malposition is present. This mechanism is important to understand and to be addressed to prevent the persistentance of SAM after surgical myectomy. Mitral valve abnormalities that need to be addressed surgically while performing myectomy include:

(a) Abnormal chordae attaching the mitral valve or the papillary muscles to the septum or free ventricular wall.
(b) Fusion of the anterolateral papillary muscle to the free ventricular wall.
(c) Coexistence of organic disease in the mitral valve causing flail segments of one or both mitral leaflets.

There is a spectrum of abnormal papillary muscle attachments or muscle bundles seen in HCM, which can be divided into four types^8^:

(a) Anomalous muscle bundles between the interventricular septum (IVS) and ventricular apex, anterior papillary muscle (APM), or posterior papillary muscle (PPM)
(b) APM or PPM adhesion to the IVS and left ventricular wall
(c) Accessory muscles that originated from the APM, PPM or IVS that insert directly into leaflets
(d) Anomalous chords attached to IVS.

### Myocardial bridges

The presence of myocardial bridges and tunnels causing compression of one or more major coronary artery is a potential cause of myocardial ischemia in HCM^9,10^. This can be easily diagnosed by pre-operative selective coronary angiography or cardiac CT scan. Myocardial bridging is often a benign condition, however, some reports have suggested a distinct association between myocardial bridging and more severe symptoms, ventricular arrhythmias, and sudden death in children with HCM. This is explained by the fact that myocardial bridges will result in coronary microvascular compromise. This will result in reverse remodeling, that may precipitate malignant arrythmia and progress to myocardial fibrosis. The adrenergic drive enhances systolic compression of the tunneled LAD artery to a sufficient degree to disturb blood flow. This is not limited to the systolic phase, but also extends into the diastolic phase, which is critical to myocardial perfusion. However, all these elements are of uncertain value and need to be revealed by randomized clinical trials to know the value of de-tunneling of myocardial bridges in HCM and its effect on the long-term outcome.

## Septal myectomy

### General overview and surgical indications

Myectomy remains the primary treatment option for most patients with severe drug-refractory heart failure symptoms, and it is becoming one of the safest open heart procedures when performed in experienced centers (mortality, 0.3%). Septal myectomy could be performed via transaortic approach, transmitral approach^11^, transapical approach^12^ as advocated by the Mayo clinic group, or a combination of all these approaches^13,14^. Additional procedures on the mitral valve are described, such as plication of the deformed and elongated mitral valve leaflets, excision of chordae with reimplantation of artificial chords, and mobilization and reorientation of the papillary muscles^11,15^. In the pediatric population, the changes seen in HCM are different and more severe, requiring careful and specific strategies for its surgical correction^43^.

The current indication for surgery is for symptomatic HCM patients with resting or provoked gradient 30–50 mmHg despite maximally tolerated medical treatment. Alcohol septal ablation (ASA) could be considered as an alternative for surgical myectomy for patients for whom surgery is contraindicated, but its effectiveness is uncertain for patients with marked hypertrophy (>30 mm)^16,17^. Septal ablation is more widely available worldwide than septal myectomy, however there are no randomized clinical trials comparing the two approaches. It was observed that the LOVT gradient is somewhat higher after ASA (average 20 mm Hg) versus myectomy (average <10 mm Hg). Myectomy is more effective than ASA in the presence of massive septal hypertrophy, which may be accompanied by midventricular obstruction. In high-volume centers that offer both myectomy and ASA, it has been observed that young patients, with massive septal hypertrophy, experienced more complete relief of symptoms after myectomy than after ASA. In older patients, who may have a lesser degree of hypertrophy, the symptomatic outcomes of the two procedures were similar. The incidence of ventricular arrythmia is higher in ASA compared to myectomy, which suggest that inducing myocardial infarction, as opposed to removal of the muscle, is responsible for these arrythmias. Transparency and reporting of an institution’s outcome are essential so that all patients can make an educated decision on the choice of procedure^18–21^.

Patients with HCM can have symptoms caused by diastolic dysfunction, small cavity size or myocardial ischemia due to small vessel disease or myocardial bridges, as well as arrhythmia. However LVOTO remains one of the most important causes of symptoms and determinent of prognosis. In our experience, surgery offers the best chance of permanent gradient and symptom relief and should be the preferred option in obstructive young HCM patients, especially when atypical pathophysiological features contribute to the obstruction^22^. The assessment of diastolic dysfunction is important and has a possible role in predicating symptom resolution post myectomy. Isoproterenol challenge can be utilized to guide decision-making and prognostication in patients with occult latent obstruction^23^.

### Historical perspectives

**Figure 4.**
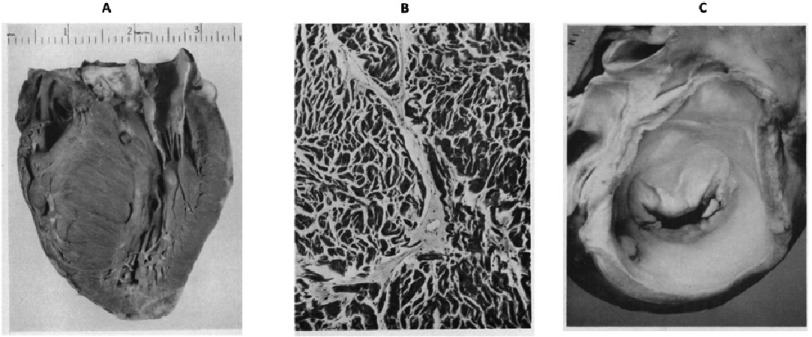
Pathologic images specimen from a report by Dr. Robert Donald Teare published in Britsh Heart Journal in 1958. These demonstrate: (a) localized hypertrophy of the interventricular septum, (b) disordered muscle fibers with variations in size of the fibers, (c) abnormal anterior mitral valve leaflet.^39^

The septal myectomy operation was introduced in the late 1950s and had a substantial evolution over time with the advancement of knowledge, imaging, and surgical techniques to relieve LVOT obstruction. Brock made the initial description of subaortic muscular hypertrophy in the absence of aortic stenosis in 1957^38^. This was followed by the description of Teare who reported an autopsy series of young patients with sudden death and asymeterical hypertrophy (Figure 4)^39^. In 1958, Goodwin described for the first time the surgical management of septal hypertrophy which was performed by Cleland in London, UK. Only a small amount of hypertrophied muscle was excised, but it was enough to produce symptomatic relief^40,41^.

In 1960, Morrow At the National Heart Institute in Bethesda introduced his technique utilizing parallel incisions into the hypertrophied septum followed by excision of the resulting bar of septal muscle using a transortic approach. Kirkilin and Ellis in the Mayo Clinic operated on two patients using a combined transaortic and transventricular approach. Cooley and associates advocated mitral valve replacement to relieve outflow tract obstruction in patients with HCM but it left patients with all the hazards of mitral valve prosthesis. However, it is well known now that in HCM patients with mitral regurgitation secondary to SAM, the mitral regurgitation resolves by myectomy alone in >95% of patients. Messmer introduced the concept of an extended septal myectomy for obstructive HCM in 1994 by having additional resection carried out the junction between septum and both the lateral wall and posterior wall at the midventricular level^42^.

### Extended septal myectomy using tailored transaortic approach

Our preferred approach for septal myectomy is the transaortic approach in almost all the cases^1^. We approach mitral valve when there is concomitant mitral disease that is not associated with HCM. Detailed conceptualization of the normal anatomy of the fibrous trigones and the changes that occur in the setting of HCM is fundamental for achieving optimal obstructive relief.

**Figure 5.**
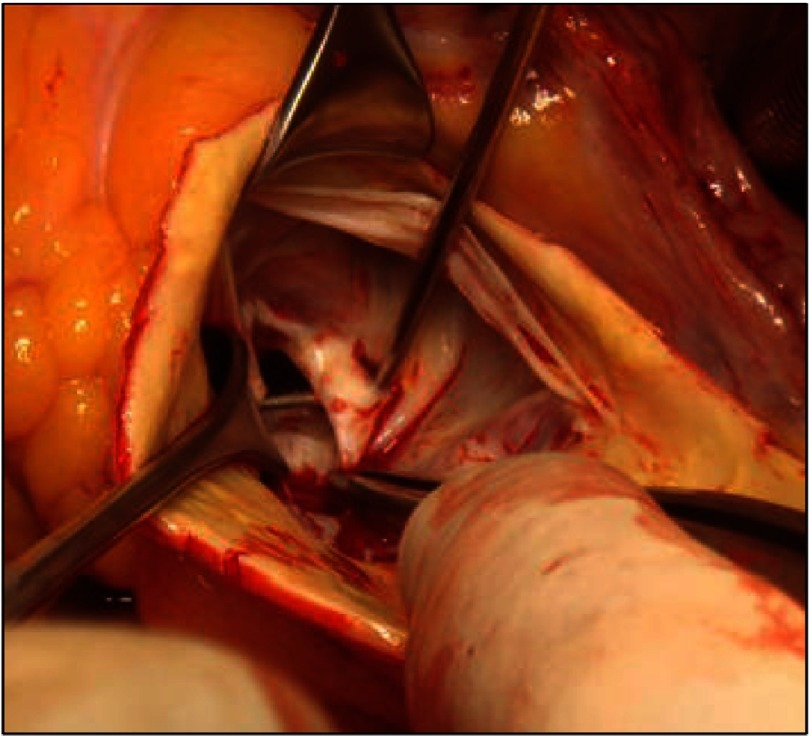
Release of the left fibrous trigone by blunt dissection, with peeling of shelf-like fibrous adhesions.^4^

We use antegrade blood cardioplegia in the aortic root or via selective coronary perfusion as our preferred method of myocardial protection. The LVOT is widely exposed through a hockey stick incision in the ascending aorta extending into the noncoronary sinus. The fibrous trigones are exposed and mobilized (Figure 5). Blunt dissection using a nerve hook releases the shelf-like obstruction and its extension onto the septum or anterior mitral leaflet, taking care not to injure the conducting tissue or the mitral valve apparatus.

**Figure 6.**
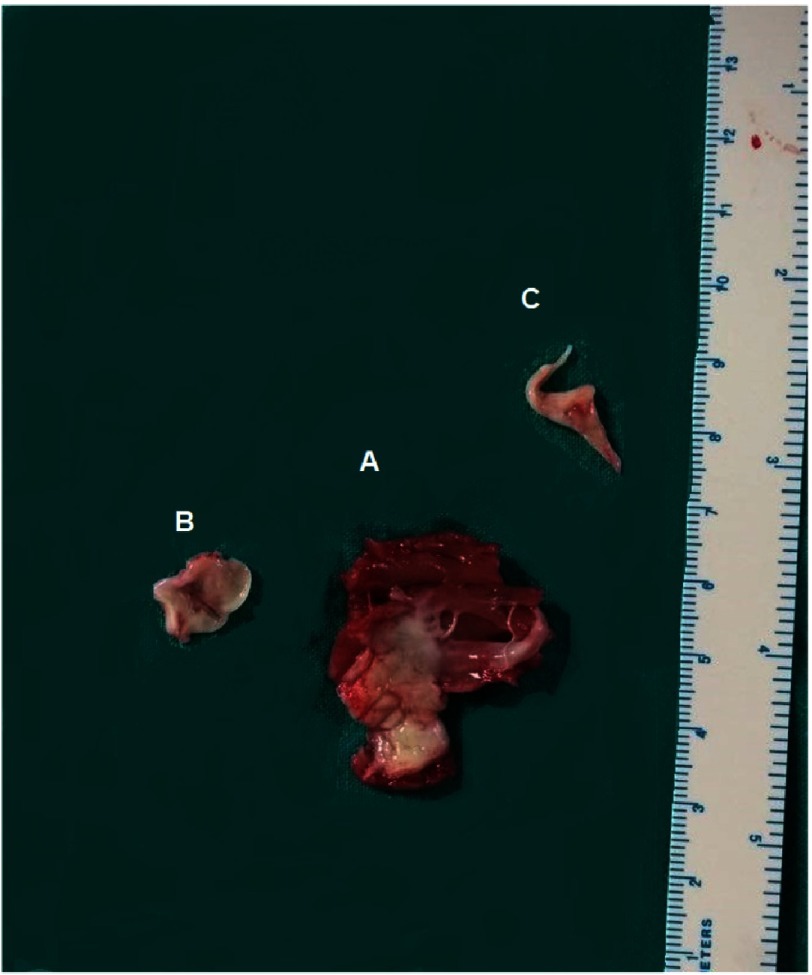
Myectomy specimen (A) illustrating the extent of the resected muscle, the released shelf like fibrous tissue on the left trigone (B), and the resected accessory chord attached to the interventricular septum.

**Figure 7.**
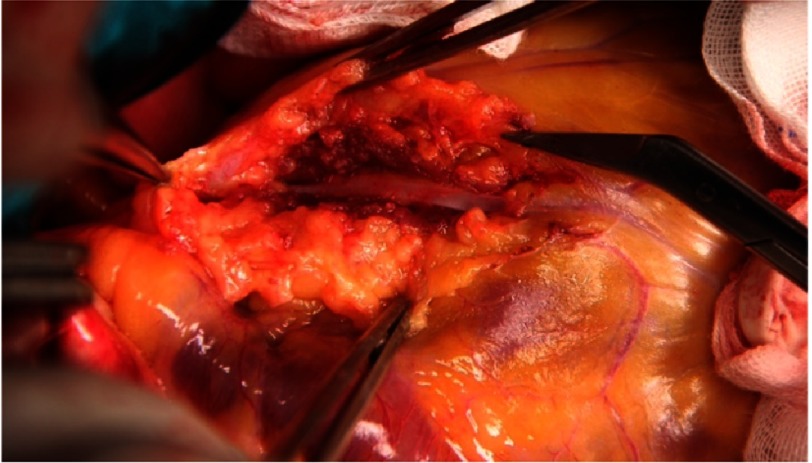
Myocardial bridge: De-tunnelling of the left anterior descending artery.^1^

The septal myectomy incision is performed starting about 1 cm from below the aortic annulus from the region of the left fibrous trigone, extending clockwise a few millimeters to the left of the membranous septum. The incision in the septum is extended into the ventricular cavity, developing a plane parallel to the endocardial surface to a level below the insertion of the papillary muscles. The thickness of the excised muscle is guided by the perioperative echocardiographic measurements. The width of the myectomy is determined by the vertical incisions, which should diverge laterally into the depth of the ventricular cavity to include all the subendocardial fibrous thickening and the three obstructive muscle bands. The fused papillary muscles are mobilized away from the septum and any accessory chords attached to the septum divided (Figure 6). In patients with myocardial bridges or tunnels causing compromise of the coronary microcirculation, surgical “de-tunnellings” are performed (Figure 7).

**Figure 8.**
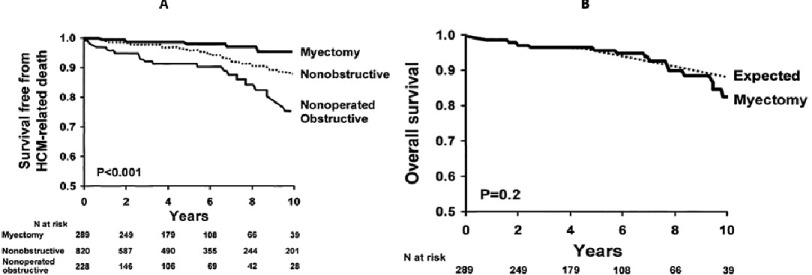
Long-term results after myectomy. (A) survival free from HCM-related death among patients in three HCM subgroups: surgical myectomy, non-operated with obstruction, and nonobstructive. (B) Survival free from all-cause mortality after surgical myectomy for obstructive hypertrophic cardiomyopathy compared with the age- and gender-matched general U.S. white population.^28^

Measurement of LVOT gradient post myectomy is essential using transesophageal echocardiogram and, if in doubt, using direct measurements. After the separation from cardiopulmonary bypass, appropriate ventricular filling, any residual obstruction is measured. The use of inotropic drugs is to be avoided as they can exacerbate LVOT obstruction. Atrial pacing is used when the heart rate is slow to improve cardiac output, especially when there is associated diastolic dysfunction.

### Surgical outcome

LV reverse remodeling occurs in all 3 layers of the myocardium at the myectomy site and in the free wall. It was found that age, disease extent, thickness of the myectomy, and sufficient relief of LVOT are important factors associated with LV remodeling^24,25^. MRI studies showed improvement in apical and midventricular systolic twist and rotation of the apical region following myectomy with unchanged basal rotations^26^. In addition, LVOT obstruction and mitral regurgitation appear to affect mechanics of the left atrium (LA). Septal myectomy results in a significant reduction in LA volumes and strain, paralleled by an improvement in function^27^.

These anatomical and hemodynamics changes post myectomy are reflected in excellent clinical outcomes. When performed by experienced surgeons, isolated septal myectomy can now be performed with a <1% mortality. Several surgical centers published their long-term outcome post myectomy. Currently, there are data on several decades follow-up of patients undergoing septal myectomy. Survival after myectomy has been shown to be equivalent to the expected survival of an age- and sex-matched general population and superior to that observed in patients with outflow tract obstruction who did not have the operation^28^ (Figure 8). Moreover, sudden death or implantable cardiac defibrillator (ICD) discharge is reduced in patients who underwent septal myectomy compared to patient who are managed medically^29,30^. This suggests a benefit of myectomy in decreasing the risk of arrhythmia, in addition to its benefit of reliving LVOT obstruction. This could be due to the improvement of coronary circulation and subsequently less risk of myocardial ischemia and arrythmia^19,31–33^.

Despite the massive need for surgical myectomy worldwide and the strong evidence for its effectiveness, application of this technique is inadequate and very sporadic. This could be due to the lack of skills and experience with the operation, reflected in the high mortality rates of the operation reported by small volume centers. On the other hand, early and late results of the operation performed in experienced centers are excellent^34–36^.

## Conclusions and future directions

**Figure 9.**
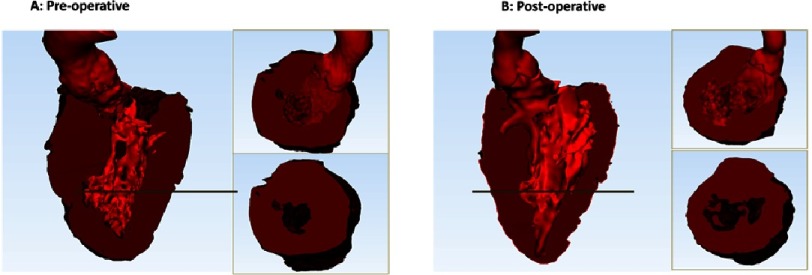
Three-dimensional modelling of the left ventricular muscle wall, systolic blood volume and LVOT pre-myectomy (A) and post-myectomy (B).

The evolution of both knowledge and experience in understanding and treating HCM encourages clinicians and scientists to further improve the outcome of current treatments. The future of HCM management will be focused on two main aspects. First, spreading the surgical experience and skills to rest of the world by having structured and dedicated surgical training on the different surgical approaches. Secondly, by improving the outcome by developing new personalized medical treatments, imaging modalities and surgical tools. 3D printing of the heart has been used for surgical planning in patients with complex congenital heart disease, cardiac tumor, and LV aneurysms, but the use of 3D printing is not well established in HCM patients. Generating 3D printed model can provide intuitive information on the LV geometry, the extent of the hypertrophied septum, location and length of the papillary muscle, and intraventricular muscle band, allowing preoperative simulation and better precision of the surgical myectomy (Figure 9)^1,37^.
